# Big Black Brain Phenomenon: Understanding Clinicoradiological Dissociation in Non-Accidental Traumatic Brain Injury in Children

**DOI:** 10.7759/cureus.8011

**Published:** 2020-05-07

**Authors:** Nitya Beriwal, Albert L Misko, Ann-Christine Duhaime

**Affiliations:** 1 Medicine, Lady Hardinge Medical College, New Delhi, IND; 2 Department of Neurology, Massachusetts General Hospital, and Harvard Medical School, Boston, USA; 3 Department of Neurosurgery, Massachusetts General Hospital and Harvard Medical School, Boston, USA

**Keywords:** hemispheric hypodensity, big black brain, non-accidental trauma, child abuse

## Abstract

Pediatric traumatic brain injury (TBI) is a major cause of concern worldwide. Non-accidental traumatic (NAT) brain injury is common in infants. Since infants may present with varied presentations post-NAT, a healthy suspicion is required for effective diagnosis. Infants with NAT and, rarely, accidental subdural hemorrhage may exhibit a clinicoradiologically dissociative presentation, with their behavior appearing to reflect better function than what becomes apparent with maturation. Injury to the developing brain can result in extensive damage consistent with the “big black brain” phenomenon, which predicts poor prognosis. Sequential magnetic resonance imaging (MRI) is important to understand insults to the developing brain for follow-up and prognostication. Pediatric traumatic brain injury (TBI) is a major cause of concern worldwide. NAT brain injury is common in infants, who may present with varied presentations post-NAT, hence, a healthy suspicion is required for effective diagnosis. Infants with NAT and, rarely, an accidental subdural hemorrhage may exhibit a clinicoradiologically dissociative presentation with their behavior appearing to reflect better function than what becomes apparent with maturation. Injury to the developing brain can result in extensive damage consistent with the “big black brain” phenomenon, which predicts poor prognosis. Sequential MRI is important to understand insults to the developing brain for follow-up and prognostication.

## Introduction

Traumatic brain injury affects up to 280 per 100,000 children worldwide, with non-accidental traumatic (NAT) brain injury being more common in infants [1]. Children may present with varied presentations post-NAT, requiring healthy suspicion for diagnosis. Infants with NAT and, occasionally, similar severe accidental injuries, may exhibit clinicoradiologically dissociative presentations, as their behavior can appear to reflect better function than what becomes apparent with maturation.

## Case presentation

**Figure 1:**
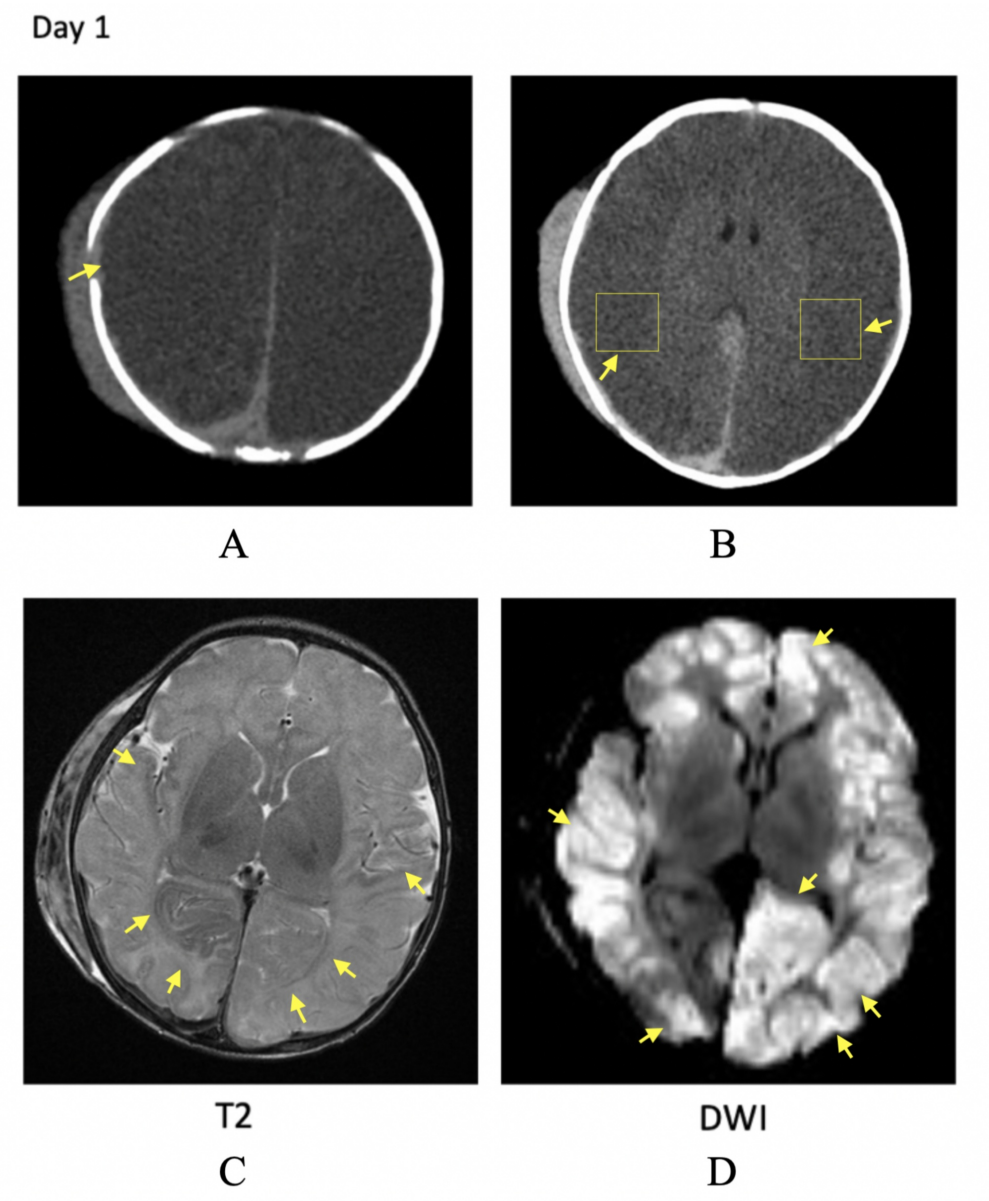
Radiological findings on day one of admission A: Head CT demonstrating displaced right parietal fracture; B: Head CT showing poor gray-white differentiation; C: MRI T2 showing diffuse pan-lobar injury; D: MRI DWI demonstrating extensive cortical diffusion restriction CT: computed tomography; MRI: magnetic resonance imaging; DWI: diffusion-weighted imaging

A 22-day-old male neonate was brought to the emergency department with a history of not waking up to feed and no history of trauma. He looked pale, lethargic, had cephalohematoma, no eye-opening, no spontaneous motor movements, stereotyped limb flexion, hypotonia, and did not cry or grimace to noxious stimulation. Head computed tomography (CT) showed a displaced right parietal fracture, subdural hematoma (SDH) bilaterally, multifocal subarachnoid hemorrhage, right subgaleal hematoma, poor gray-white differentiation, and brain edema. The patient was intubated for hypoxia secondary to hypoventilation. Post transfer to the pediatric intensive care unit (ICU), he was noted to have myoclonic movements for which levetiracetam was started, with fentanyl and midazolam drips for sedation. MRI confirmed the CT findings with an extensive abnormality on diffusion-weighted imaging (DWI). It showed cortical diffusion restriction and diffuse pan-lobar injury (Figures 1A-1D). In view of significant injury and likelihood for breakthrough seizures, long-term electroencephalogram (EEG) monitoring was started. He was found to have status epilepticus after levetiracetam load and midazolam infusion; loading with phenytoin and phenobarbital led to the cessation of seizures. After day three, no seizure activity was observed.

**Figure 2:**
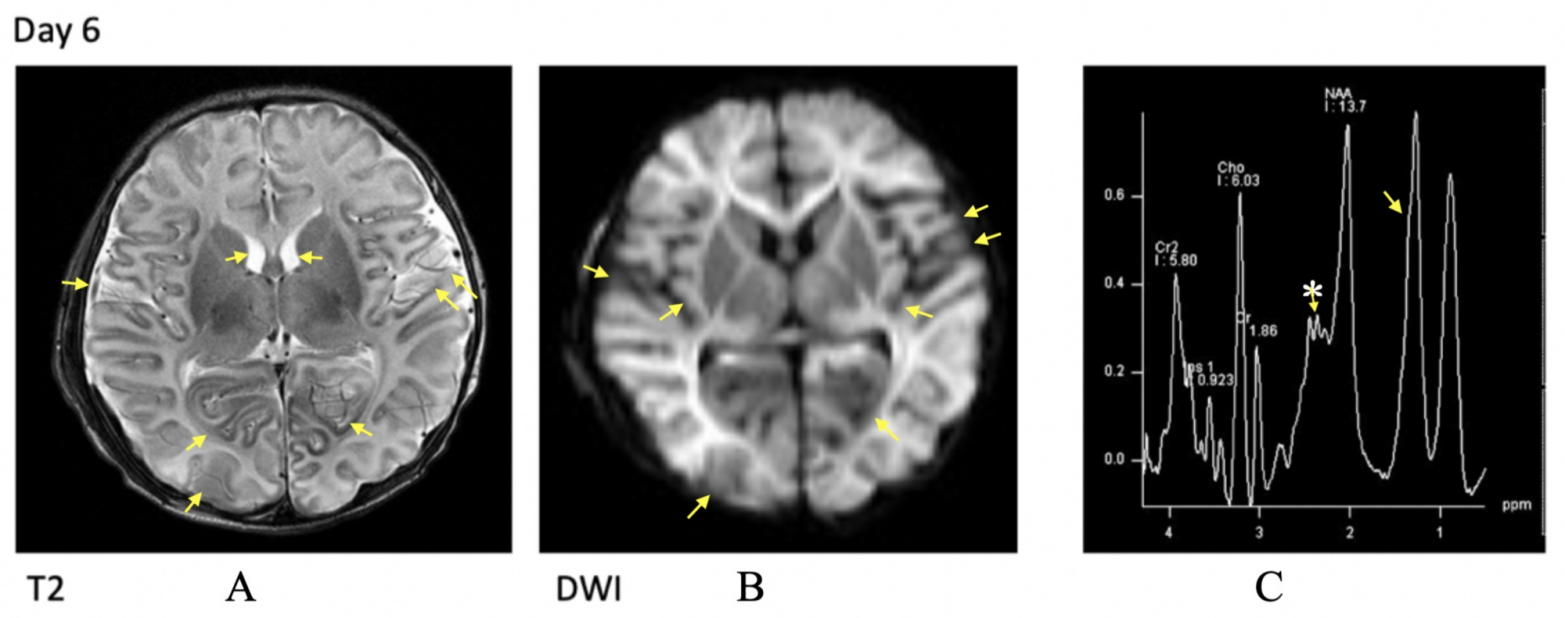
Radiological findings on day six of admission A: MRI T2 shows diffuse panlobar encephalomalacia bilaterally. Interval enlargement of ventricles seen; B: MRI DWI demonstrates abnormal diminished diffusion in cortical and subcortical areas bilaterally; C: MR spectroscopy (TE=40) shows glutamine/glutamate (Glx) shoulder (*) with lactate peak in the occipital cortex MRI: magnetic resonance imaging; DWI: diffusion-weighted imaging

Repeat brain MRI after six days showed the interval evolution of panlobar injury, diffuse panlobar encephalomalacia bilaterally, with abnormal diminished diffusion in the cortical and subcortical areas. Interval enlargement of bilateral subdural collections and enlargement of the ventricles related to diffuse involution of the brain parenchyma was noted secondary to the aforementioned encephalomalacic changes. Interval reduction in the size of extra-axial blood products and foci of intraparenchymal blood was also observed. MR spectroscopy (MRS) (TE=40) shows glutamine/glutamate (Glx) shoulder indicative of excitotoxicity with a lactate peak (Figures 2A-2C). EEG monitoring was continued with phenobarbital and sedation wean over the subsequent month.

**Figure 3:**
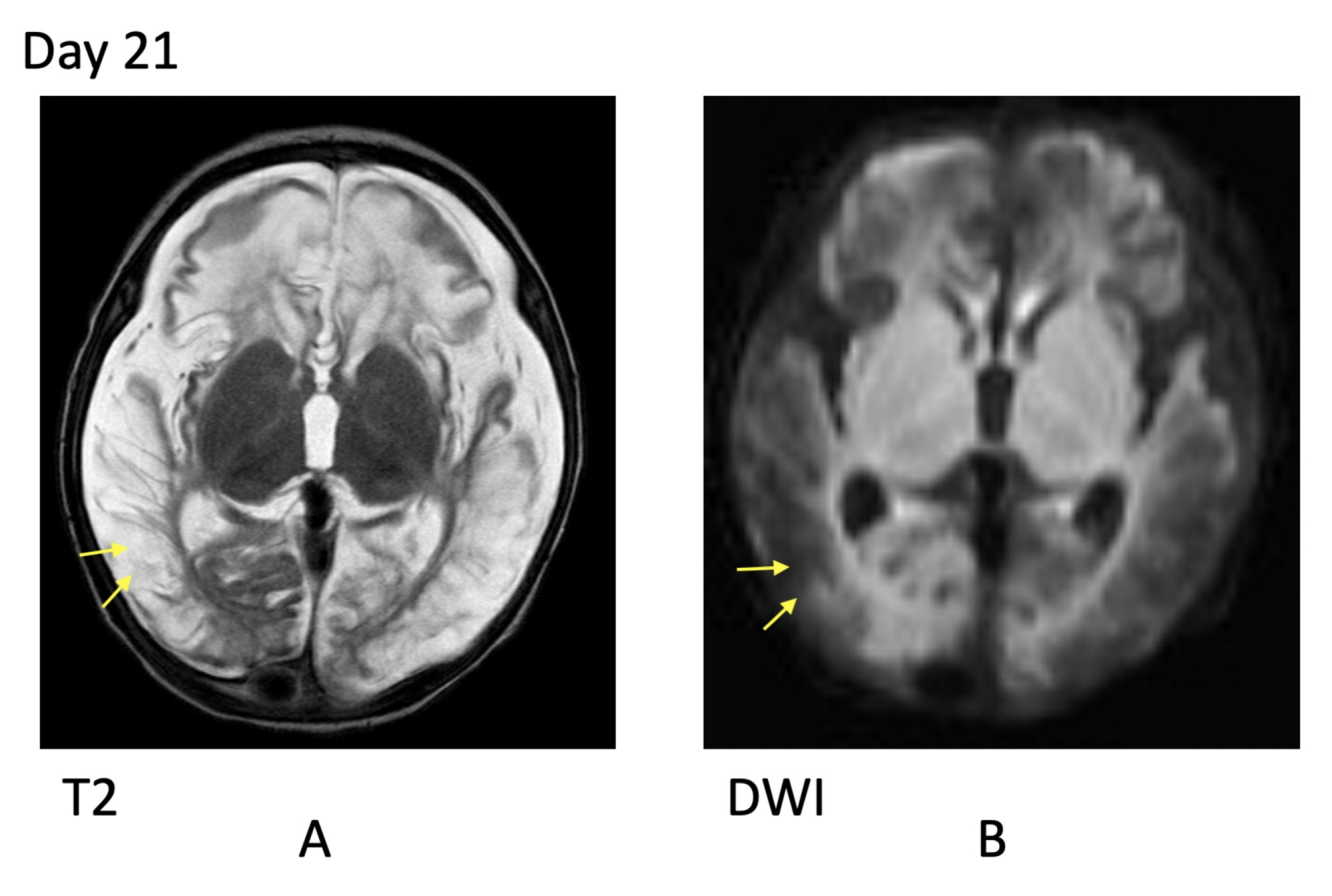
Radiological findings on day 21 of admission A: MRI T2 showing severe diffuse cortical atrophy, including the occipital cortex; B: MRI DWI demonstrates abnormal (facilitated) cortical diffusion, appearing dark, consistent with diffuse cortical encephalomalacia MRI: magnetic resonance imaging; DWI: diffusion-weighted imaging

On repeat evaluation at two months of age, his clinical examination demonstrated an increased level of wakefulness, increased spontaneous movements, and intact brainstem reflexes. Visual tracking to movement was present. Saccadic eye movements to sound were present, with a negative optokinetic reflex. He grimaced to pain with spontaneous high-pitched crying. However, the MRI showed severe diffuse cortical atrophy, including the occipital/visual cortex (Figures 3A-3B). While the exam appeared improved overall, brain MRI findings showed progression of diffuse cortical encephalomalacia consistent with the “big black brain” phenomenon, predicting poor prognosis.

## Discussion

Infants are prone to a distinctive injury pattern of developing unilateral or bilateral hemispheric hypodensity (HH) or “big black brain,” terms reflective of CT findings in the setting of acute SDH. HH is believed to reflect a decompensation of hemispheric cerebral perfusion/metabolic demand related to SDH and other stressors, including hypoventilation, hypoxia, and seizures or other electrophysiologic perturbations, although the exact pathophysiology is incompletely understood [2]. The visual tracking movements seen in our patient reflect a brain stem reflex to non-specific movement similar to the oculocephalic reflex. This pathway involves the retina, superior colliculus, and inferior olive of the midbrain and includes a complex network with the thalamus and cerebellum [3]. This fix and follow motion can occur without cortical input in the setting of brainstem recovery. The child is at risk of recurrent seizures with impairment of vision, spasticity, and minimal motor and higher cortical function [4]. Baseline and sequential MRI studies are helpful to understand insults to the developing brain, as clinical assessment in the subacute period may not be a reliable prognostic indicator.

Currently, CT is generally the initial imaging modality of choice for the assessment of acute head injury due to high accuracy in detecting fractures and hemorrhages [5]. MRI use has become more widespread in acute trauma, as it is more sensitive for parenchymal lesions and early ischemic changes [6]. Imaging features commonly seen in NAT patients with severe injuries include SDH, loss of gray-white differentiation, parenchymal intensity changes, and hemispheric swelling with midline shift (in the unilateral form), which are appropriately delineated on MRI [7]. Similar clinical and radiologic findings can occur in accidental injuries, though accidental subdural hemorrhage with a severe clinical presentation is uncommon in infants. This clinical case highlights the utility of baseline and sequential MRI follow-up in infants with NAT for better brain injury characterization, prognostication, and long-term management, as an insult to the developing brain can result in widespread damage (like the “big black brain” phenomenon), unlike the injury pattern seen in adults.

## Conclusions

An insult to the developing brain in infants can result in neurological damage like the bilateral hemispheric hypodensity or the “big black brain” phenomenon. This may result in a clinicoradiologically dissociative presentation. Therefore, baseline and sequential MRI has utility for follow-up in infants with NAT for effective assessment of their brain injury to facilitate prognostication and clinical management.
