# Correction: Big Black Brain Phenomenon: Understanding Clinicoradiological Dissociation in Non-Accidental Traumatic Brain Injury in Children

**DOI:** 10.7759/cureus.c31

**Published:** 2020-05-14

**Authors:** Nitya Beriwal, Albert L Misko, Ann-Christine Duhaime

**Affiliations:** 1 Medicine, Lady Hardinge Medical College, New Delhi, IND; 2 Department of Neurology, Massachusetts General Hospital and Harvard Medical School, Boston, USA; 3 Department of Neurosurgery, Massachusetts General Hospital and Harvard Medical School, Boston, USA

The abstract was originally included twice and this duplication was, unfortunately, not caught by our copy editors. We have removed the duplicated text.

